# Development of a new critical size defect model in the paranasal sinus and first approach for defect reconstruction—An in vivo maxillary bone defect study in sheep

**DOI:** 10.1007/s10856-022-06698-9

**Published:** 2022-10-20

**Authors:** R. Rothweiler, S. Kuhn, T. Stark, S. Heinemann, A. Hoess, M. A. Fuessinger, L. S. Brandenburg, R. Roelz, M. C. Metzger, U. Hubbe

**Affiliations:** 1 Department of Oral and Maxillofacial Surgery, Faculty of Medicine, University of Freiburg, Hugstetter Straße 55, 79106 Freiburg, Germany; 2 Stryker Leibinger GmbH & Co. KG, Bötzinger Straße 41, 79111 Freiburg, Germany; 3 INNOTERE GmbH, Meissner Str. 191, 01445 Radebeul, Germany; 4 Department of Neurosurgery, Faculty of Medicine, University of Freiburg, Breisacher Str. 64, 79106 Freiburg, Germany

## Abstract

Fractures of the paranasal sinuses often require surgical intervention. Persisting bone defects lead to permanent visible deformities of the facial contours. Bone substitutes for reconstruction of defects with simultaneous induction of new bone formation are not commercially available for the paranasal sinus. New materials are urgently needed and have to be tested in their future area of application. For this purpose critical size defect models for the paranasal sinus have to be developed. A ≥2.4 cm large bilateral circular defect was created in the anterior wall of the maxillary sinus in six sheep via an extraoral approach. The defect was filled with two types of an osteoconductive titanium scaffold (empty scaffold vs. scaffold filled with a calcium phosphate bone cement paste) or covered with a titanium mesh either. Sheep were euthanized after four months. All animals performed well, no postoperative complications occured. Meshes and scaffolds were safely covered with soft tissue at the end of the study. The initial defect size of ≥2.4 cm only shrunk minimally during the investigation period confirming a critical size defect. No ingrowth of bone into any of the scaffolds was observed. The anterior wall of the maxillary sinus is a region with low complication rate for performing critical size defect experiments in sheep. We recommend this region for experiments with future scaffold materials whose intended use is not only limited to the paranasal sinus, as the defect is challenging even for bone graft substitutes with proven osteoconductivity.

**Figure:**
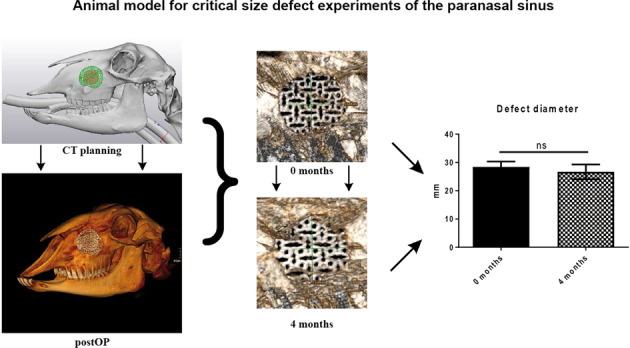
Graphical abstract

Graphical abstract

## Introduction

**Fig. 1:**
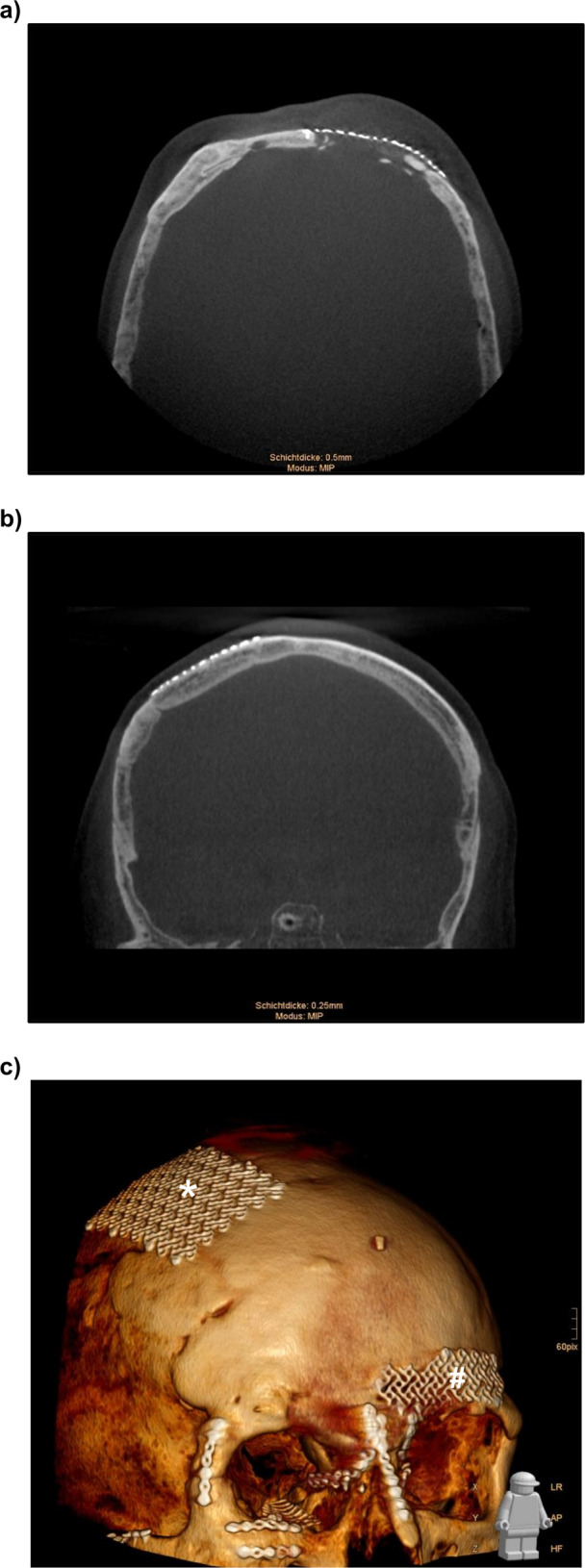
CT scan of a patient 20 years after fracture treatment of the skull [(**a**): axial slice of a frontal sinus fracture; (**b**) coronal slice of a fracture of the parietal bone]. No proper healing can be observed in the frontal sinus area, using only a simple covering with a titanium mesh. **c** 3D reconstruction of the same CT scan showing the exact location of the two fracture regions in the frontal [#] and parietal [*] region

The midfacial and frontal area (paranasal sinuses) are the most endangered regions for fractures of the human skull. 5–15% of all craniofacial fractures related to trauma are localized in the frontal sinus region [1]. 85% of all polytraumatized patients show fractures in the midfacial region [2]. Adequate repositioning of even the thin bones is essential to restore an appropriate cosmetic appearance and to avoid visible deformities of the facial contours. Remaining defects caused by limited healing compared to other body regions lead to scarring and soft tissue retraction, which may result in neuropathic pain (Fig. 1) [3].

Over the past 20 years, several surgical techniques revolutionized sinus fracture repair. Endoscopic transnasal approaches allow treatment of simple median fractures of the frontal sinus without the need of an external incision [4, 5]. In certain cases, a closed reduction of isolated anterior frontal sinus fractures using temporarily percutaneously inserted screws shows adequate reduction results with no visible scarring [6]. The use of resorbable plates can avoid the need for a re-operation for plate removal especially in children. Their indication however is limited to low-stress non-load-bearing areas of the middle and upper craniofacial skeleton [7].

In patients where direct surgical treatment with correct positioning of the fracture fragments is not possible due to concomitant injuries, or in cases with large remaining defects, reconstruction with alloplastic materials often remains the method of choice. Materials used are mainly PMMA, ceramics, titanium, PEEK or porous polyethylene [8–10]. The materials used do not have the ability to become ingrown in living tissue, a characteristic an ideal bone graft substitute should provide [11].

In vivo investigation of new materials and scaffolds has become increasingly important with technological advances before being translated into clinical practice, as not all questions can be answered with in vitro testing. Animal models used allow evaluation in loaded or unloaded situations over a long period of time and different tissue qualities [12]. In bone reconstruction a distinction is made between “large size bone defects” and “critical size bone defects”. While large size defects can heal during lifetime of an animal, this is not the case with critical defects, a definition first made by John Schmitz in 1985 [13]. The diameter required to create critical size defects depends on several variables, such as animal species, sex, age, ratio of bone thickness to defect diameter, defect location, removal of periosteum or even the health status [14, 15].

Experiments with critical size defects in the maxillofacial region of sheep are usually performed in the mandible or the calvarium. Studies of the mandible comprise three to five wall defects on the lateral side or bottom side of the mandible, which on the one hand prevents from instability of the mandibular bone and opening to the oral cavity on the other hand [16–20]. Studies in the calvarium perform circular defects via an extraoral approach [21]. The defects themselves are left open or covered with titanium meshes [22]. However, to date, no representative defect model exists for reconstruction of paranasal sinus defects, especially in the midface region.

The main objective of the present work was to establish a standardized and safe critical size defect model in the anterior maxillary sinus of sheep as representative region for repair of paranasal sinus defects. Reconstruction was performed with a proven osteoconductive titanium scaffold to verify the characteristics of the defect on the one hand and the suitability of the scaffold for the midface region on the other [23]. A defect covered with a titanium mesh served as a control.

## Materials and methods

### Animals and Ethical approval

The experiment was done in six female Merino sheep older than three years. An ethical approval for the experiment was obtained from Regierungspräsidium Freiburg (No: 35-9185.81/G-19/121). Detailed animal and scaffold data are listed in Table 1.

**Table 1:** Animal & scaffold data

Sheep number	Ear Tag	Anterior maxillary wall	Scaffold / mesh design
S1	17350	Right	CPC-filled scaffold
		Left	Empty scaffold
S2	46698	Right	CPC-filled scaffold
		Left	Empty scaffold
S3	13817	Right	Titanium mesh
		Left	CPC-filled scaffold
S4	60770	Right	CPC-filled scaffold
		Left	Titanium mesh
S5	46802	Right	Titanium mesh
		Left	CPC-filled scaffold
S6	0450041	Right	Empty scaffold
		Left	CPC-filled scaffold
*N* = 12		*N* = 12; *N* = 6 right; *N* = 6 left	Titanium mesh: *N* = 3 Empty scaffold: *N* = 3 CPC-filled scaffold: *N* = 6

### Anaesthesia, analgesia and antibiotic therapy

Sheep were sedated with midazolame (0.2–0.5 mg/kg) and ketamine (20 mg/kg) twenty minutes before general anaesthesia. For induction of general anaesthesia animals received propofol (2–6 mg/kg) intravenously. Maintaining of anaesthesia was done with the use Isoflurane (1% in 0.6–1l/min oxygen).

For analgesia Fentanyl (5–10 µg/kg/h) combined with ketamine (10 mg/kg/h) was administered intravenously preoperatively. Postoperative pain relief was performed using a Fentanyl dermal patch (100 µg/kg for 3 days) combined with Carprofen subcutaneously (4 mg/kg/24 h for at least 3 days).

A perioperative antibiotic treatment regime was chosen for infection prophylaxis. Preoperatively, 2 g of Ceftriaxone were administered intravenously direct before operation. After surgery postoperative treatment consisted of an intramuscular injection of Amoxicillin with long half-life (Duphamox; 15 mg/kg), repeated once after 48 h.

### Scaffold design and manufacturing

All meshes and scaffolds were planned, and designed patient specifically based on a CT scan taken preoperatively of the individual sheep. Empty defects of the control groups were covered with a 3D-printed, 0.6mm thick commercially pure Titanium Mesh showing a pattern that corresponded to commercially available Meshes (Dynamic Mesh, Stryker Leibinger).

**Fig. 2:**
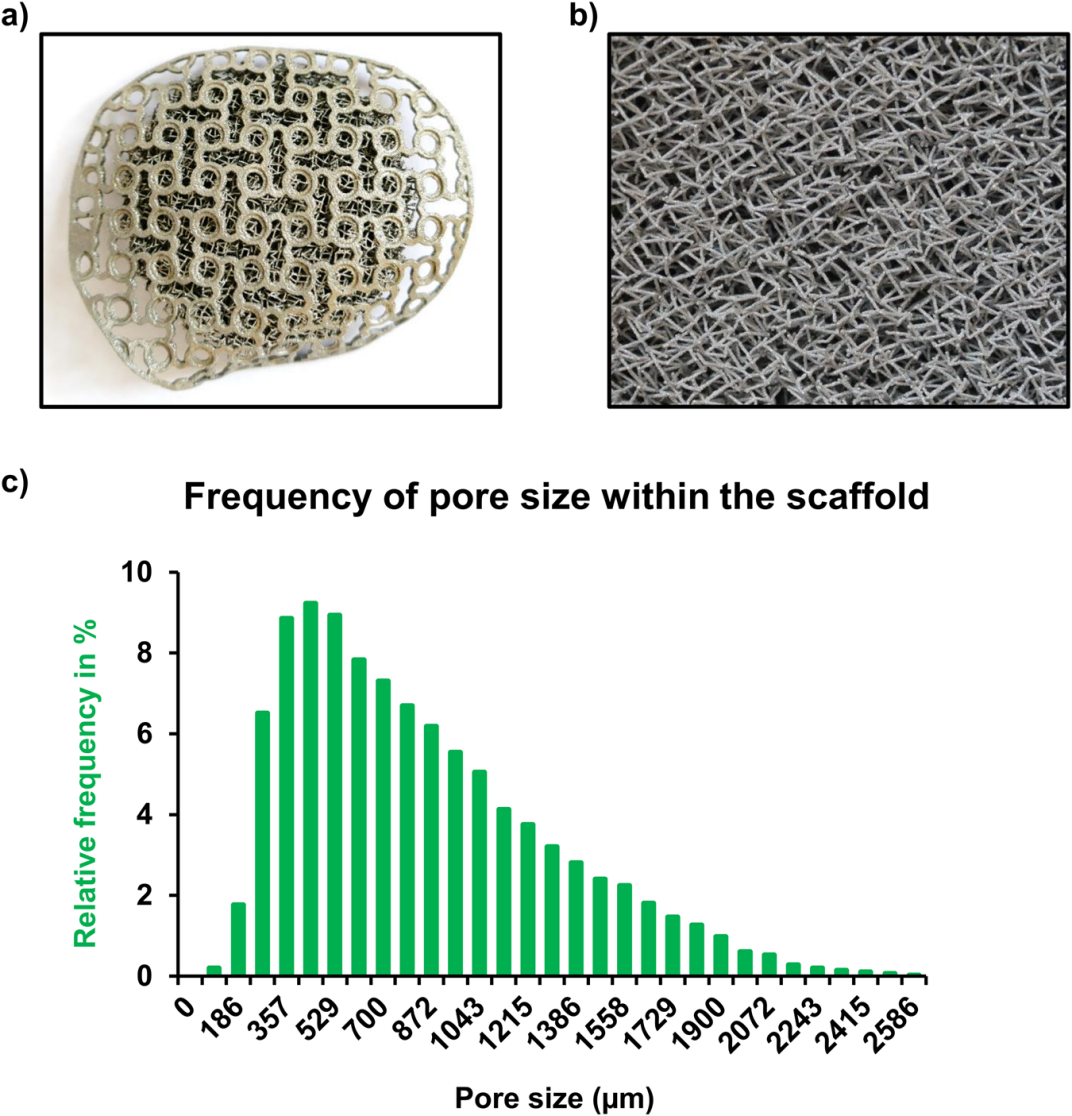
**a**, **b** Examplary images of the scaffold [(**a**): from top {overview}; (**b**): from bottom {high magnification}]. **c** Relative frequency of occurrence of pores sorted by size within the scaffold

Scaffolds were designed consisting of a mesh for fixation on the outer part and an adjacent porous body with a multidirectional, stochastially distributed pattern and variable pore sizes with a peak at 450 and 1150 µm allowing bone ingrowth. The porous body had the form of a cone to increase the surface to the remaining bone (Fig. 2).

Porous bodies of the scaffolds were either left untreated or filled intraoperatively with a calcium phosphate bone cement paste (INNOTERE Paste-CPC) containing β-TCP particles with a size of 100–250 μm (both INNOTERE, Radebeul, Germany) [24]. CPC paste was administered using a special 3D-printed filling mold.

### CT scans and image analysis

CT scans were initially performed for planning and manufacturing the 3D patient specific designed and printed cutting guides and implants. Further CT scans were carried out directly after surgery and after sacrification. Data was stored as DICOM stacks. Image analysis was done with ImpaxxEE R20 Software (Agfa HealthCare N.V., Mortsel, Belgium) and RadiAnt DICOM Viewer [25]. CT densities were measured in Hounsfield Units (HU) at five different predefined spots with a diameter of 4 mm. An overview of the spots is given in Appendix Fig. 7.

### Push-Out-Tests

Push-Out tests were performed using one half of the explanted scaffold / mesh that was not used for histological analysis. Each sample was mounted on a Zwick-Roell compression machine after removal of the fixation screws (Zwick Z010 / PM04690). An increasing force was applied to the mounted samples. Forces measured were Maximum Force (F_max_) and First Drop of Force seen before breakage (F_drop_).

### Histology

Histological samples were fixed in 4% neutral buffered formalin. After fixation dehydration was done by the use of an ascending alcohol series. Dehydrated samples were afterwards embedded in Methyl methacrylate (CAT # 8.00590, Sigma-Aldrich Chemie GmbH, Taufkirchen, Germany). PMMA blocks were cut into slides with a thickness of 1mm. Staining of slides was done using an adapted Levai-Laczko protocol [26].

Bone ingrowth from the lateral side was measured as maximum length parallel to the scaffold / mesh surface using ImageJ (ImageJ Version 1.53; NIH, USA) [27]. All calculations were done in triplicates from three consecutive histological slides.

### Statistical analysis

Statistical analysis was done using GraphPad Prism Software Version 8.3.0 (GraphPad Software, San Diego, USA) and SPSS Statistics V22.0 (IBM, Armonk, USA). Data are presented as mean±SD, comparisons and statistics were calculated using Mann–Whitney-Test, Tukey’s multiple comparisons test and Kruskal Wallis test. Results were considered statistically significant when *p* < 0.05.

## Results

### Surgical procedure

**Fig. 3:**
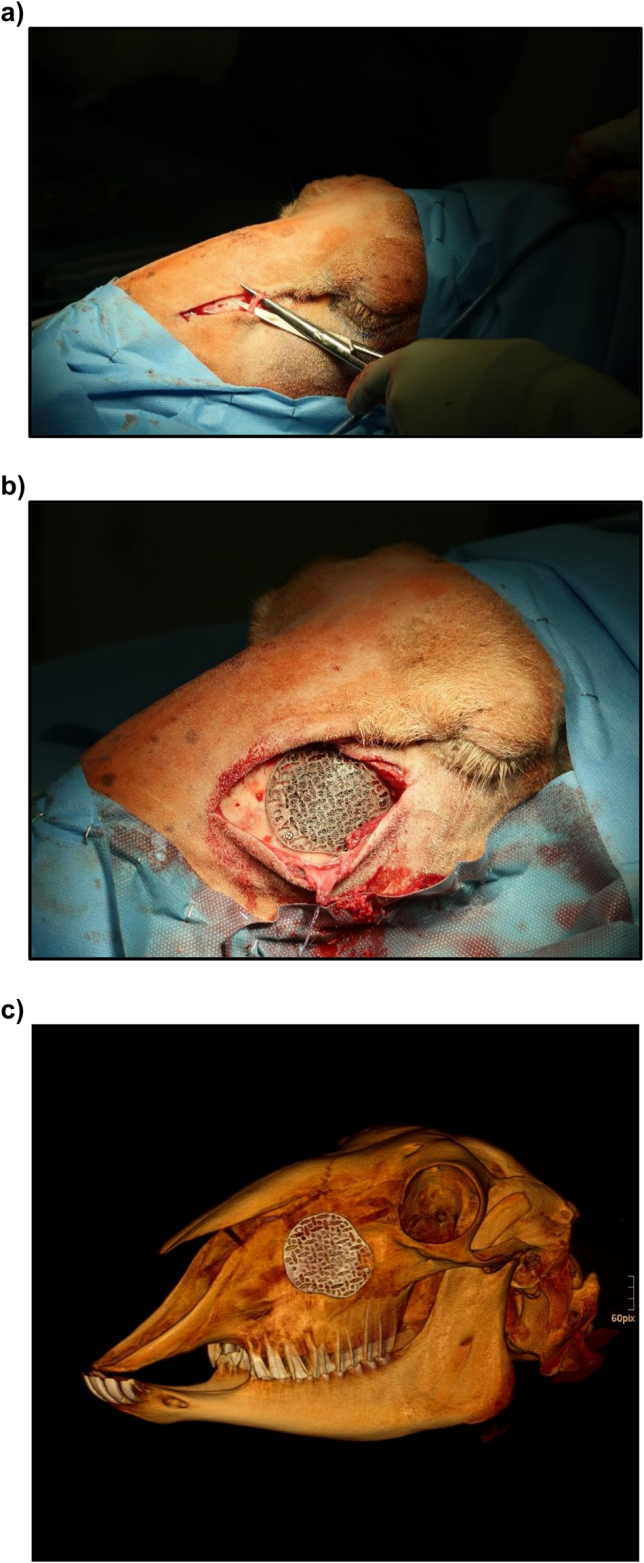
**a**, **b** Intraoperative exemplary image after preparation of the angular vein and insertion of an empty scaffold into a 2.4 cm defect in the anterior wall of the maxillary sinus. **c** Postoperative CT scan showing correct placement of the scaffold

The defect was placed on both sides in the anterior wall of the maxillary sinus. To avoid contamination with intraoral bacteria an infraorbital extraoral approach with a L-shaped skin incision was chosen. After disinfection of the fur and skin incision the angular vein was identified and ligated with resorbable suture (Fig. 3a). The approach was completed with a sharp incision and detachment of the periosteum. A 3D manufactured cutting guide was attached to the anterior wall of the maxillary sinus using three 5mm screws (Stryker: CAT # 56-15905; Ti6AL-4V). The circular >2.4 cm defect itself was created using a trephan and a craniotome. The Schneiderian membrane could be spared, small cracks in it were adapted with resorbable suture (polyglactin 910 4-0). The resulting 12 defects were filled with a scaffold or covered by a preformed 3D-printed titanium mesh serving as control group (empty scaffold: *n* = 3; CPC-filled scaffold: *n* = 6; mesh: *n* = 3) (Fig. 3b+c). Fixation of the scaffolds or the meshes was done using five 5mm screws per mesh / scaffold. The temporary detached masseter muscle was readapted at the infraorbital rim using resorbable suture. Wound closure was finally done in a multi-layered technique with resorbable continuous suture pattern in the deep layers (polyglactin 910 2-0) and a non-resorbable vertical mattress suture as final non-resorbable skin closure (polyamide 2-0). Non-resorbable stitches were removed seven days after surgery.

### Postoperative phase and presentation of final results

The postoperative phase was uneventful. All sheep recovered well after surgery, no wound dehiscence occurred, and sheep were euthanized as initially planned four months after surgery. Post-mortem CT scans revealed all scaffolds and meshes being still in place as inserted during operation, except of one CPC-filled scaffold (1/6).

**Fig. 4:**
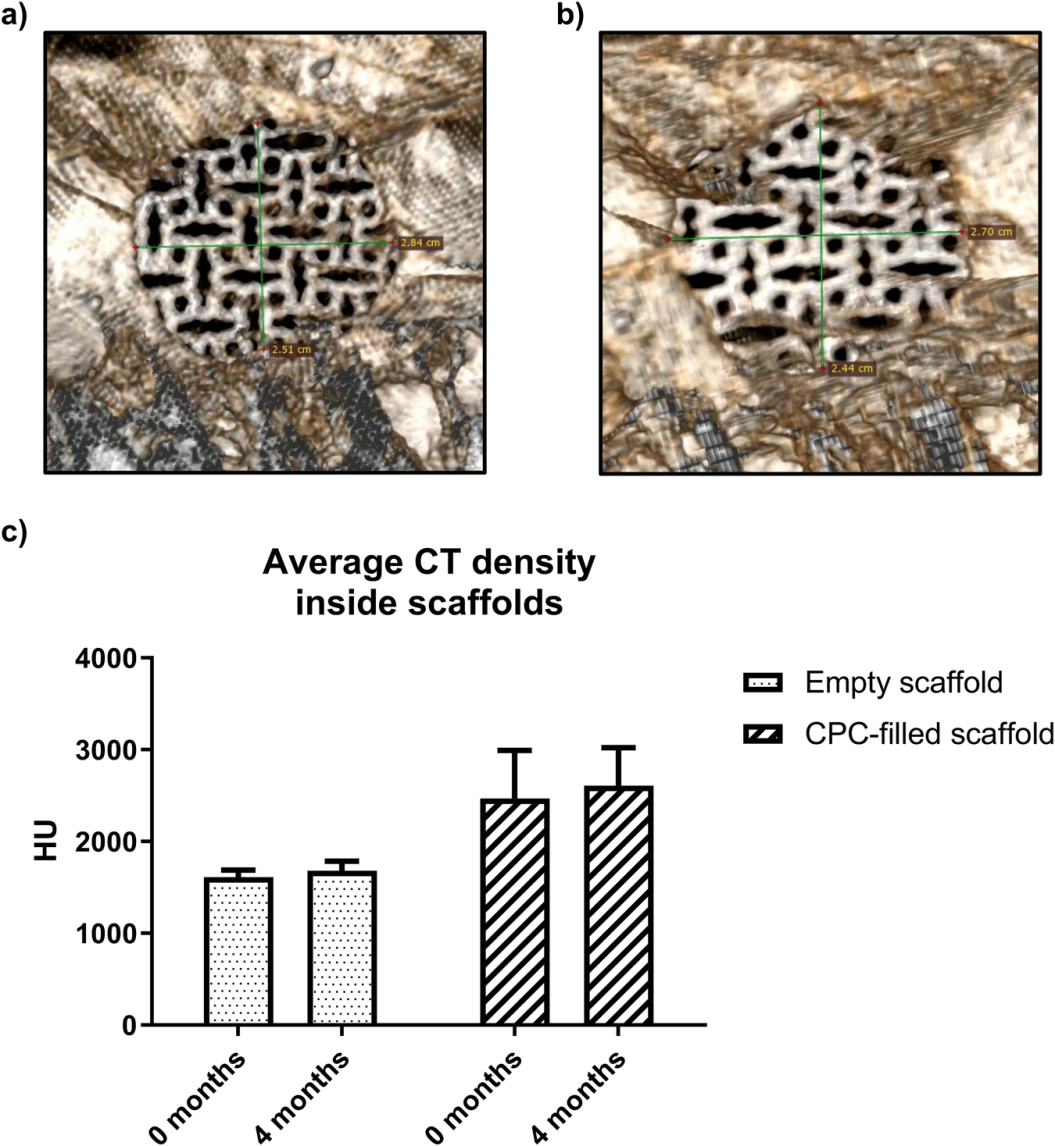
CT scans of the defect of the control group confirming the successful creation of a critical size defect [(**a**): direct postoperatively; (**b**): CT after euthanization]. **c** Results of the average CT density measured at five points inside the scaffolds. Values between the different points in time do not differ significantly. Higher denitites of the CPC-filled group are caused by the CPC paste

The defect of the control group covered with titanium mesh, revealed only minimal shrinkage during the investigation period. The diameter of the defect was >24 mm in all sheep, confirming the successful creation of a critical size defect [26.7 mm ± 2.4 (t = 4 months) vs. 28.4 mm ± 1.74 (t = 0)] (Fig. 4a+b). CT density analysis showed minimally higher but not significantly different CT densities inside the empty scaffold at 4 months after euthanization in comparison to the direct postoperative control (0 months: 1612.0 HU ± 137.0; 4 months: 1680.0 HU ± 113.7; *p* = 0.78). The average density of the scaffold filled with CPC was higher than that of the empty scaffold due to the filling, but also did not differ significantly between the different study time points (0 months: 2467.0 HU ± 535.0; 4 months: 2607.0 HU ± 437.0; *p* = 0.62) (Fig. 4c).

**Fig. 5:**
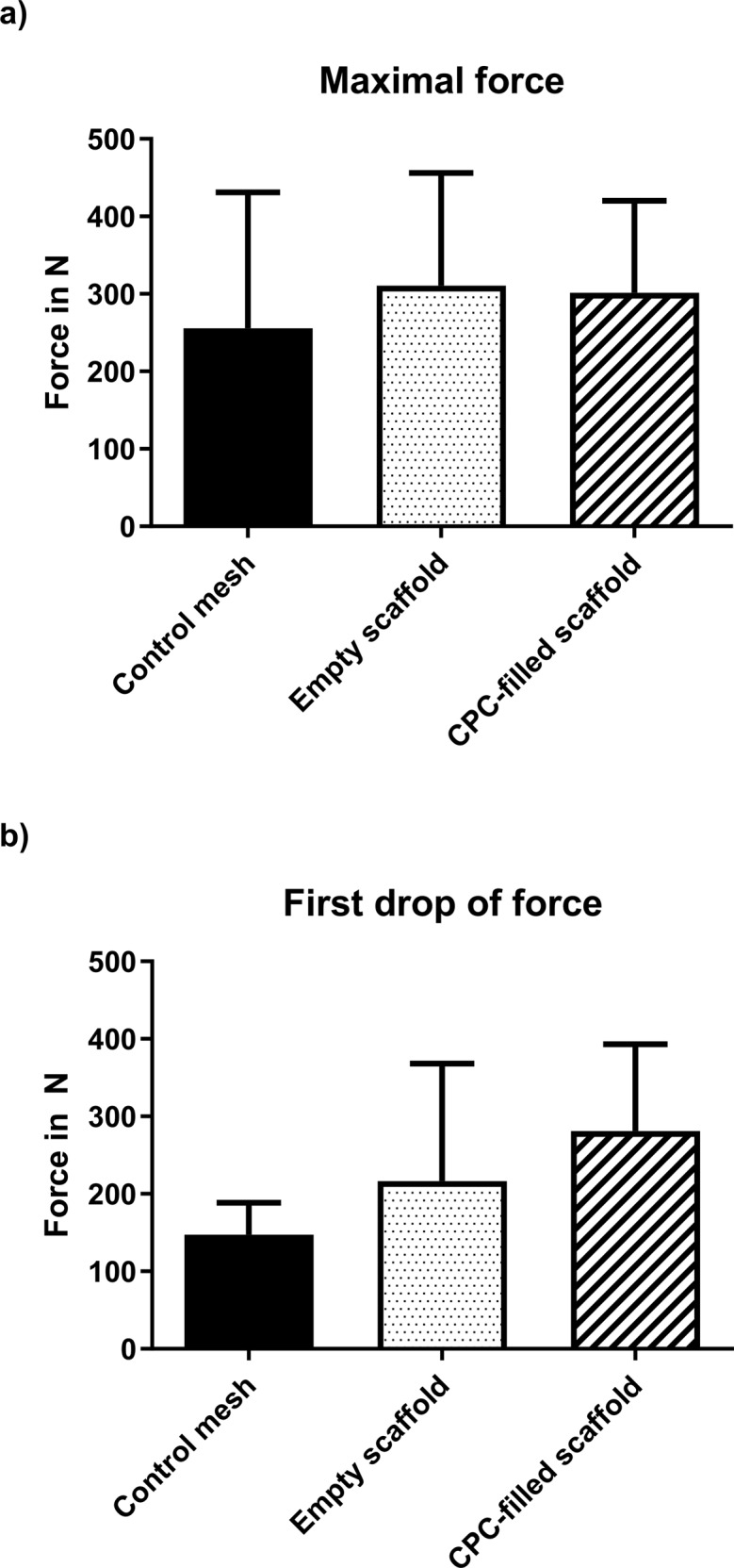
Push-out tests showing only minimal differences among the different experimental setups [(**a**): Maximum force; (**b**): First drop of force]

Push-out test performed directly after euthanization with half of the transplant could not identify statistically significant differences in the maximal force measured (Control mesh: 255.8 N ± 175.2; empty scaffold: 310.7 N ± 145.1; CPC-filled scaffold: 308.7 N ± 104.0; *p* = 0.49–0.58). The measurement of the force beginning with a sudden drop before breakage showed similarly not statistically significantly different results (Control mesh: 147.5 N ± 40.9; empty scaffold: 216.3 N ± 151.7; CPC-filled scaffold: 281.0N ± 112.1; *p* = 0.06–0.51) (Fig. 5).

**Fig. 6:**
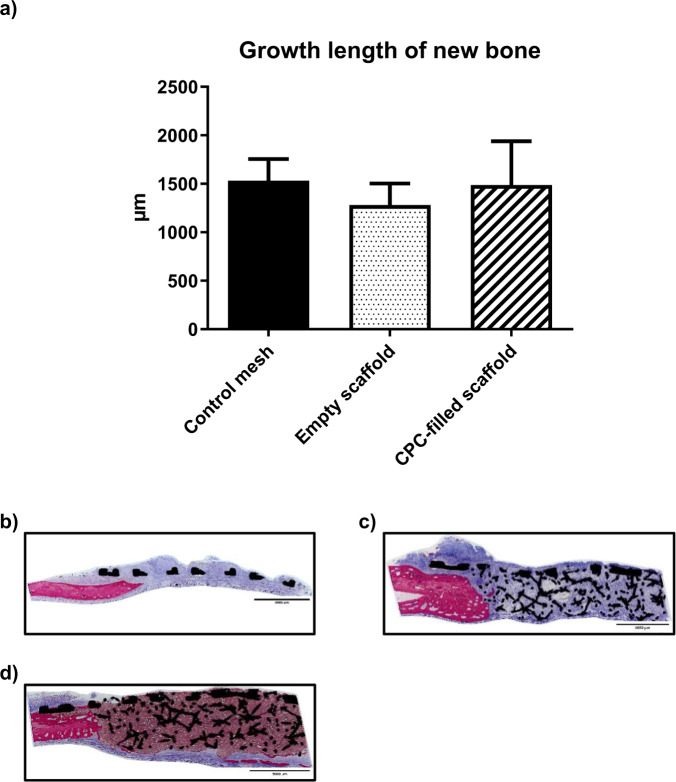
Histological analysis shows no bone ingrowth into the scaffolds at all [(**a**): average length of newly formed bone from the lateral side. Exemplary cross sections: (**b**) control group; (**c**) empty scaffold; (**d**) CPC-filled scaffold]

Final histologic analysis showed no bone ingrowth into either the empty or CPC-filled scaffolds. Bone growth could only be detected in the small empty space between resection borders and the scaffold. Lengths measured from lateral were: Control mesh: 1531.0 µm ± 223.8; empty scaffold: 1281.0 µm ± 221.7; CPC-filled scaffold: 1487.0 µm ± 452.4. Results did not differ significantly (*p* = 0.14–0.31) (Fig. 6).

## Discussion

In the present study, we describe for the first time the successful realization of a critical-size defect model in the anterior maxillary wall of sheep as a representative area for reconstruction of paranasal sinus defects. Reconstruction of the defect with an osteoconductive titanium scaffold developed for cranioplasty was not successful due to increased demands of the defect on the implant material.

In the last decades bone tissue engineering has developed to one of the most important research fields in trauma and craniomaxillofacial surgery. Standardized animal models have been established to translate engineered products into a clinical setting before product launch [28]. The most frequently used models are calvarial defects, long bone and segmental defects, partial cortical defects and cancellous bone defects [29]. Large size bone defects are distinguished from critical size defects, defects that do not heal during animal’s lifetime [13].

The use of sheep as laboratory animal has increased in the recent decades and has become one of the most used animals in orthopaedic research [30]. The animals show weight comparable to humans, are docile, easy to handle, easy to house and widely available [31]. In contrast to orthopaedic research, where standardized defect and fracture models with low complication rates are widely available for sheep, only a few cranial and maxillofacial models have been described for this species. The most commonly performed defect is the calvarium defect. The calvarial region is safe and has been shown to have an extremely low complication rate [21, 32, 33]. Defects in the mandibular region comprise three to five wall defects on the lateral side or bottom aspect avoiding an opening to the oral cavity, a compelling condition first described by Söderholm et al. [34]. Continuity interrupting defects are less common and carry a high risk of failure.

Our approach describes an alternative type of defect in the midface region to test scaffold materials for repair of paranasal sinus defects. Using a simple extraoral approach as variation of the classic Weber-Fergussin approach, a good overview of all structures in the maxillary region can be achieved while avoiding contamination with intraoral pathogens [35]. The bone is easy to reach and the only important anatomical structure that needs to be ligated is the angular vein, which is not important for the blood supply of the face. The infraorbital nerve, another important anatomic structure, does not need to be sacrificed because the infraorbital foramen is located much more anteriorly adjacent to the nose. A defect size of ≥2.4 cm in diameter is large enough to be considered a critical defect size. Total operating time for a bilateral defect can be limited to <1.5 hours because no important structures need to be preserved and the bone in this region is not very thick.

Comparable surgical approaches have been described only in a few isolated cases in literature before, none of them for defect surgery. Aim of these studies was a maxillary sinus augmentation model for implant studies rather than the fabrication of a defect model. Gutwald et al. and Alayan et al. both reported a low complication rate for their approach and proved their approaches were safe for augmentation surgery. The Schneiderian membrane, which is very tough in most cases, had a low rupture rate, which was an important prerequisite for preventing contamination with pathogens originating from the maxillary sinus [36, 37]. Estraca et al. described an intraoral approach to the anterior maxillary wall. However, he complained of a limited overview of the surgical site and needed an additional buccal skin incision of 2 cm to overcome this problem [38].

In the present study, defect reconstruction using an osteoconductive titanium scaffold developed for cranial reconstruction did not show any ingrowth of bone into the scaffold at all [23]. In the case of the CPC-filled scaffolds, the situation was further challenging by the fact that the necessary degradation of the filling material did not take place during the observation period. The material concept, which envisaged that the β-TCP particles would be rapidly degraded by physicochemical processes and that the hydroxyapatite-forming matrix phase would be degraded in the course of bone remodelling, could not be achieved [39]. These results are somewhat surprising at first glance but can be explained by local conditions and have to be attributed to the bone itself. While skull bones or bones of other body regions generally consist of a cortical and cancellous part, the walls of the paranasal sinus are very thin and consist only of a cortical part missing the osteogenic and osteoconductive more potential cancellous bone (*Appendix* Fig. 8) [40, 41]. The underlying Schneiderian membrane of the maxillary sinus, which also has a slight osteoinductive potential, could not compensate for this problem although small areas of new bone were detected at the interfaces in some histological cross sections [42, 43]. The circumstances just described make the established defect more challenging for scaffold materials compared to other defect regions. It is reasonable to assume that successfully tested scaffolds in this region will perform in other regions as well.

One possible solution to overcome this problem with limited / no bone ingrowth into the scaffold could be surface modification of the scaffolds used. Optimization of SLM sintering protocols, such as reducing the laser power during the melting process, increasing the scanning interval, or the thickness of the cut layers can raise surface roughness of titanium scaffolds which helps to improve bone ingrowth [44, 45]. Surface coating with inorganic materials such as hydroxyapatite can optimize osteoconductive properties of the scaffold and contributes to a high degree of tissue ingrowth and vascularization [23, 46].

In addition to surface and structure optimization of the scaffold the introduction of stem cells or endothelial cells into the scaffold could be a promising approach to be considered in future experiments [47]. Additional supplement of growth factors such as BMP2 or BMP7 could further enhance their effect [48, 49].

To our knowledge, this is the first description of a model of critical size defects in the anterior wall of the maxillary sinus of sheep. Based on our results we suggest this model as standard approach for paranasal sinus defect experiments in sheep as it provides a low complication rate and good overview over the surgical site with simultaneous short operating times. The identified defect poses a greater challenge to scaffold materials in contrast to other locations. It can be assumed that scaffolds that have been successfully tested in this region are very likely to perform well in other defect regions.

## Conclusion

The anterior wall of the maxillary sinus is a suitable region for performing critical size defects in the paranasal sinus of sheep. Contamination of the wound with intraoral pathogens can easily be avoided by a simple external approach, which offers a good overview of the surgical site. A circular defect size of ≥2.4 cm is sufficient to achieve a critical size defect. The Schneiderian membrane can be preserved, which offers additional protection from bacteria of the maxillary sinus. Due to local factors, the defect itself presents a greater challenge to scaffold materials than other craniofacial regions.

## Figures and Tables

**Fig. 7:**
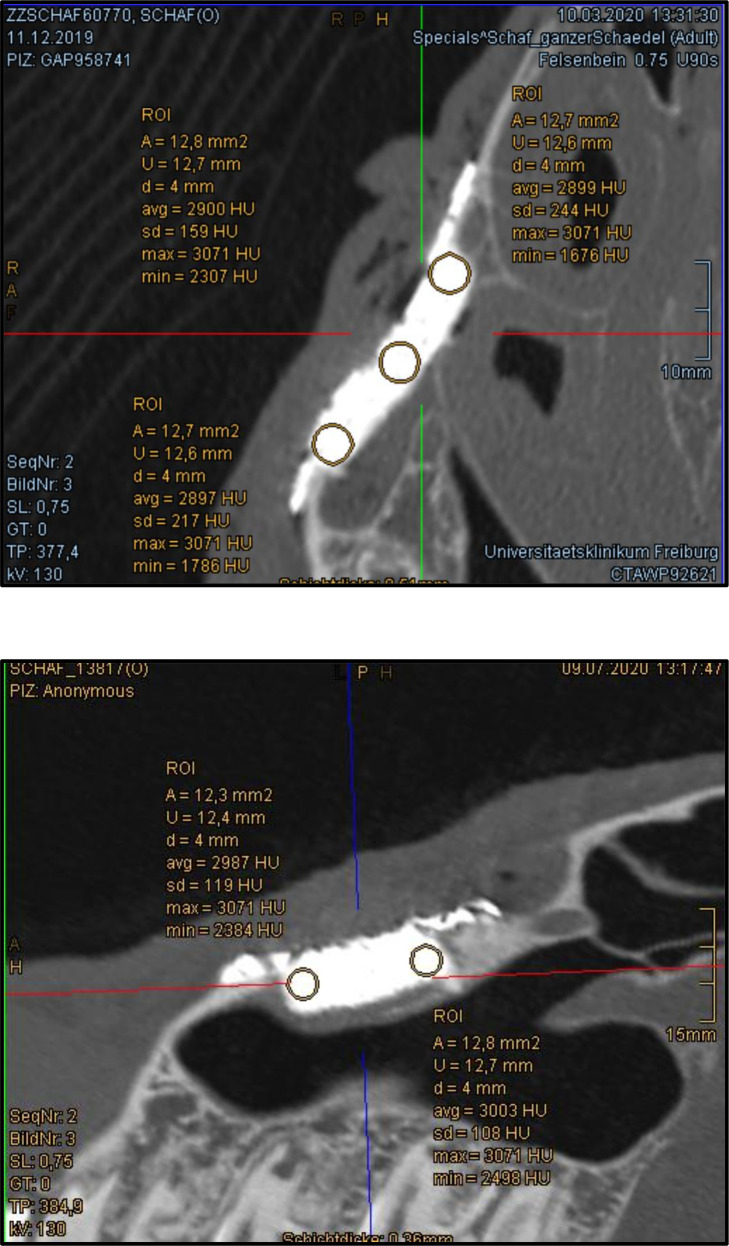
Exemplary images of the spots used for investigation of density analysis. Four spots were positioned at the border of the scaffold, one was placed in the middle of the scaffold

**Fig. 8:**
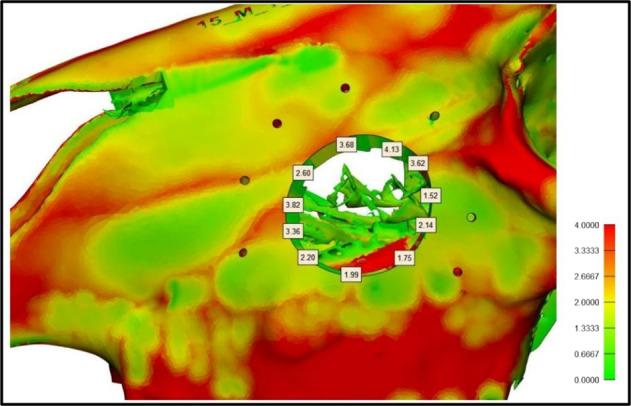
Preoperative virtual planning of the future defect in the anterior wall of the maxillary sinus; Heatmap showing the average thickness of the bone in mm

## Appendix

Figures 7 and 8
